# Unity Makes Strength: A Review on Mutualistic Symbiosis in Representative Insect Clades

**DOI:** 10.3390/life9010021

**Published:** 2019-02-25

**Authors:** Rosario Gil, Amparo Latorre

**Affiliations:** 1 Institute for Integrative Systems Biology (I2SysBio), Universitat de València/CSIC. Calle Catedrático Agustín Escardino, 9, 46980 Paterna (Valencia), Spain; 2 Departament de Genètica, Universitat de València. Calle Dr. Moliner, 50, 46100 Burjassot (València), Spain; 3 Área de Genómica y Salud, Fundación para el Fomento de la Investigación Sanitaria y Biomédica de la Comunidad Valenciana (FISABIO). Avenida de Cataluña 21, 46020 València, Spain

**Keywords:** endosymbiosis, genome-reduction syndrome, consortium, primary endosymbiont, secondary endosymbiont, *Buchnera*, *Sulcia*, *Tremblaya*, symbiotic replacement, minimal genomes

## Abstract

Settled on the foundations laid by zoologists and embryologists more than a century ago, the study of symbiosis between prokaryotes and eukaryotes is an expanding field. In this review, we present several models of insect–bacteria symbioses that allow for the detangling of most known features of this distinctive way of living, using a combination of very diverse screening approaches, including molecular, microscopic, and genomic techniques. With the increasing the amount of endosymbiotic bacteria genomes available, it has been possible to develop evolutionary models explaining the changes undergone by these bacteria in their adaptation to the intracellular host environment. The establishment of a given symbiotic system can be a root cause of substantial changes in the partners’ way of life. Furthermore, symbiont replacement and/or the establishment of bacterial consortia are two ways in which the host can exploit its interaction with environmental bacteria for endosymbiotic reinvigoration. The detailed study of diverse and complex symbiotic systems has revealed a great variety of possible final genomic products, frequently below the limit considered compatible with cellular life, and sometimes with unanticipated genomic and population characteristics, raising new questions that need to be addressed in the near future through a wider exploration of new models and empirical observations.

## 1. Introduction

### 1.1. Brief History of Endosymbiosis and Its Importance in the Evolution of Eukaryotes

In nature, species do not live alone but interact with others, and their interactions can have a strong impact on their evolutionary histories. Symbiosis, broadly defined as “living together” [1], is nowadays acknowledged as one of the main forces shaping life in our planet, as the evolutionary fate of the members of a steady association is mutually dependent, leading in some cases to co-cladogenesis. According to the fitness effects on the two (or more) symbiotic partners, such relationships can be referred as mutualism when both species increase their fitness, parasitism when one species increases its fitness while the fitness of the other is adversely affected, and commensalism when one partner is increasing its fitness without affecting the other one. Yet, there are no clear barriers among these possible interactions and rather we encounter a continuum that many species can transit along their life history.

Eukaryotes from numerous clades maintain mutualistic relationships with prokaryotes, mainly bacteria [2]. Depending on the location of the symbiont with respect to the host cells, it is referred as endosymbiosis when the prokaryote symbiont lives inside a specialized eukaryote cell, called bacteriocyte, and ectosymbiosis when the symbiont lives on the host’s body surface. Finally, according to the degree of dependence, the association can be obligate (or primary) and facultative (or secondary). Again, there are no clear barriers between these two categories, and facultative bacteria can become obligate under special circumstances (reviewed in [3]).

In addition to the two canonical endosymbioses that were the origin of mitochondria and chloroplasts, stable mutualistic associations have evolved frequently and independently in numerous eukaryotes groups [2]. Most of such symbioses have a biochemical basis. In the case of animals, their metabolisms are heterotrophic and many nutrients must be obtained from external sources. Thus, endosymbiotic bacteria can be used as factories for the provision of essential biomolecules that are lacking in the host diet.

Most early studies about symbiosis between bacteria and eukaryotic hosts focused on mutualistic and obligate insect–bacteria endosymbiosis. The work of scientists like the zoologist Umberto Pierantoni and the embryologist Karel Šulc at the beginning of the past century revealed that this type of relationship was widespread in many insect groups [4,5]. They recognized the bacteriome (at that time called mycetome) as an organ dedicated to keeping microorganisms inside the insect body. Inspired by these pioneering studies, Paul Buchner dedicated a good part of his scientific career to describe and decipher the meaning of the diversity of beneficial associations between insects and endosymbiotic bacteria. Among the examples presented by him in his seminal book published in English in 1965 [5], some are still being analyzed nowadays, providing new insights on this kind of intimate association, and will be the focus of this review.

### 1.2. Similar Unbalanced Diets but Different Host-Symbiont Associations

Insects represent around 85% of animal diversity, and some estimations indicate that around 15% of them maintain an endosymbiotic relationship with bacteria that, due to the strict dependence between host and symbiont, are called primary (P-) endosymbionts [6,7]. The establishment of these associations is conceived as one of the key factors of the evolutionary success and diversification of this animal group. Even though new data are accumulating, most studies concentrate on nutritional and physiological aspects (summarized in [3,8]), because one characteristic feature of most mutualistic symbiosis, as already stated by Buchner, is that the hosts feed on specialized diets lacking essential nutrients, which must be supplied by their allied bacteria [5]. The association is mutualistic because each host provides its endosymbiont(s) with a stable environment with a permanent resource provision, but it is also obligate. As a consequence, most of these bacteria cannot be cultured outside their hosts and, for this reason, should be referred to as “*Candidatus*” [9], although this criterion has not always been taken into account. In most cases, although the cognate insects are considered independent species, a single species name has been given to all bacteria associated with large insect clades, with each insect species presenting a given bacterial strain. For simplicity sake, along this review, the full name of the bacterial species will only be indicated the first time it appears in the text (without the “*Candidatus*” statement), and we will normally refer to the bacterial genus when there is only one species described.

In the genomics era, research on bacterial endosymbionts focused on the same limited number of insect lineages that had been previously used as models to define the evolutionary and molecular aspects of prokaryote-animal symbioses. Most of them belong to the order Hemiptera, the sap-sucking insects of suborders Sternorrhyncha and Auchenorrhyncha being the most widely screened. Aphids, psyllids, white flies, and mealybugs (Sternorrhyncha) feed on phloem sap that is rich in carbohydrates, but deficient in nitrogen compounds. They have established mutualistic relationships with different P-endosymbionts (*Buchnera, Carsonella, Portiera*, and *Tremblaya*, respectively), and in many cases with the help of other bacteria to meet the host’s needs [7]. Among them, the symbiotic systems found in aphids and mealybugs will be the object of Section 2 and Section 4. Auchenorrhyncha include clades feeding on phloem (planthoppers, treehoppers, and most leafhoppers) and xylem (cicadas, spittlebugs, and primitive leafhoppers), the latter diet being the one considered ancestral [10]. Xylem is less nutritious than phloem, containing mostly minerals and inorganic compounds but being poor in organic nitrogen and carbohydrates. Most surveyed Auchenorrhyncha (both xylem- and phloem-feeding lineages) harbor *Sulcia* as P-endosymbiont, but it is almost always accompanied by another symbiotic partner, maintaining complex associations that were referred to by Buchner as “a fairyland of insect symbiosis” [4] that will be later unraveled (see Section 3). Other insects from different orders, also with restricted diets, have been studied, including the mammalian blood-feeding tsetse flies (Diptera) and lice (Phthiraptera), whose endosymbionts *Wigglesworthia* and *Riesia*, respectively, provide the B-complex vitamins lacking in blood, and plant feeding weevils (Coleoptera) such as *Sytophilus*, whose endosymbiont, *Sodalis pierantonius* (in honor to the pioneering work of Umberto Pierantoni), provides tyrosine for the development of the cuticle. The case of carpenter ants (Hymenoptera) and cockroaches (Blattodea) was striking because, as omnivorous animals, they feed on a complex diet but still harbor obligate endosymbiotic bacteria. The genome sequencing of their respective endosymbionts, *Blochmannia* and *Blattabacterium*, showed that their obligate dependence is related to nitrogen storage and recycling during the stages in their life cycle in which their diet cannot provide it [11,12].

Comparative genomics of the first sequenced endosymbiont genomes allowed for the determination that, during the integration process from free-living to endosymbiont, the bacteria underwent drastic genetic, phenotypic, and biochemical changes, which could be detected by comparison with free-living relatives. The changes observed are a consequence of the small effective population size undergone during the vertical transmission of the endosymbiont to the offspring, and the absence of horizontal gene transfer to compensate genetic drift [13]. Some changes, such as the accumulation of small deleterious mutations, increase in the number of non-synonymous substitutions, accelerated evolutionary rates, the loss of many regulatory functions, and the loss of mobile elements (mainly insertion sequences) are common to all long-term endosymbionts sequenced so far; others have been proven to be more lineage-specific, including an increase in high A+T content (quite general but with some interesting exceptions that will be described later), genome stasis, and plasmid-mediated gene amplification. However, the most relevant characteristic, shared by all bacteria engaged in an intracellular lifestyle, is the genome size reduction by gene loss [2]. For this reason, this phenomenon is known as “the genome-reduction syndrome” [3].

Due to the huge amount of information accumulated during the last two decades, it would be impossible to summarize all cases in a single review. For this reason, we will focus on selected model insects harboring bacterial endosymbionts for which there is abundant literature since the very early times of the field of symbiosis, and in which different degrees of interaction with the hosts have been identified within close relatives. These examples will help us to detangle the molecular aspects of bacteria–insect symbioses from a genomics and evolutionary perspective.

## 2. Aphids as the First Defined Symbiotic Model: *Buchnera* and Its Multiple Partners

### 2.1. Historical View of the Aphid-Buchnera Systems

In honor of Buchner, Paul Baumann and his group gave the name of *Buchnera aphidicola* to the endosymbiotic bacteria (an Enterobacteriaceae within the class Gammaproteobacteria) found in the aphid *Schizaphis graminum* [14]. Although it was not given the status of *Candidatus*, it cannot be cultured outside its host, so the bacterium was defined based on its shape, localization within the aphid host, and 16S rRNA gene sequence. Since then, all the P-endosymbionts of aphids have been named *B. aphidicola*, even though they have coevolved with their hosts belonging to divergent aphid lineages, thus having different species names. It was the second bacterial endosymbiont of insects characterized at that time, after *Blattabacterium cuenoti*, endosymbiont of cockroaches [15]. Treatment with antibiotics to reduce or eliminate the bacterial endosymbionts caused abnormal growth, lack of reproduction, and premature death, confirming the obligate status of the endosymbiont [16]. The hosts have developed mechanisms to regulate, maintain, and transmit their symbionts; thus, as all other obligate endosymbionts, *Buchnera* is strictly vertically transmitted from mother to offspring. The works by Baumann and his disciples in the USA on molecular characterization of *Buchnera* [17] were complemented by the metabolic characterization of the aphid–bacterium system, thanks to the research performed by the team led by Angela Douglas in the UK [16], and culminated with the sequencing of the first *Buchnera* genome (in fact, the first sequenced endosymbiont genome) in the laboratory of Hajime Ishikawa in Japan [18].

Phylogenetic studies of aphids and their endosymbionts showed that *Buchnera* is a root cause of the diversification of aphids, indicating that the ancient acquisition of a *Buchnera* free-living ancestor (about 200 million years ago (MYA)) was followed by coevolution of host and symbiont, and the specialization of the host to different feeding niches [19]. Thus, each extant aphid species harbors a *Buchnera* strain suited for providing the specific nutrients that are deficient in its diet, mainly essential amino acids and some vitamins. Now that paired host/endosymbiont genomes are becoming available, it is recognized that the host is the one controlling the P-endosymbiont production of the required nutrients in a finely tuned metabolic complementation, mainly by performing some final steps in the corresponding biosynthetic pathways [20,21,22]. Amazingly, some of the genes involved in these functions are of bacterial origin, acquired through horizontal transfer from environmental bacteria other than the current obligate symbionts (revised in [23]). It has been proposed that the eventual acquisition of bacterial genes by the host genome is another factor contributing to the extreme genome reduction found in long-term P-endosymbionts [24,25].

The first five aphid species from which their *Buchnera* genomes were sequenced belong to three different subfamilies: Aphidinae (*Acyrtosiphum pisum* and *S. graminun)*, Eriosomatinae (*Baizongea pistacia*), and Lachninae (*Cinara cedri* and *Cinara tujafilina)* [18,26,27,28,29], providing information of the genomic changes undergone by *Buchnera* diversifying since the origin of aphids. The comparative genomic analysis revealed an extreme case of evolutionary stasis with nearly perfect gene order conservation. However, important differences in genome size were detected, *Buchnera* BAp (from *A. pisum*) and *Buchnera* BCc (from *C. cedri*) having the largest and the smallest genomes (610 and 402 kb), respectively. Their chromosomal stasis allowed for the reconstruction of the evolutionary history of losses occurring in the different lineages from the *Buchnera* ancestor, which should have contained at least 646 genes (the sum of all genes found in the five strains) [29]. At present, the genomes of *Buchnera* from 24 different aphid species have been sequenced and deposited in GeneBank, revealing that they maintain the gene order fossil, and the metabolic analysis of 18 of them is accessible through SymGenDB [30].

### 2.2. From Facultative to Co-Obligate Symbionts: The Establishment of Microbial Consortia

Occasionally, aphids harbor secondary (S-) symbionts, facultative bacteria that coexist with *Buchnera*. The genome sequencing of the pea aphid *A. pisum* showed that its immune system is compromised as it has lost the complete IMD pathway that acts against Gram-negative bacteria [20]. This could have been decisive in recognizing *Buchnera* as a non-pathogen (whether as a cause or a consequence of the interaction), but it also implies that other bacteria, mostly Gram-negative, can enter the insect without an effective immune barrier. In fact, all bacterial S-symbionts described in aphids up to now are Gram-negative or do not have a cell wall, as is the case of *Spiroplasma* [31]. They can be found in different host tissues (in their own bacteriocytes, in sheath cells, free in the haemolymph, etc.), and are normally vertically transmitted from mother to progeny, although horizontal transmission is also possible [32,33]. As they are not found in all strains nor in all individuals of a population, they are considered non-essential to the host. However, positive effects have been proven in some cases, such as rescuing the host from heat damage, providing resistance against natural enemies (as parasitoids and fungi), participating in host specialization, inducing phenotypic variation, or even causing insect color change) [31,34,35]. Because facultative symbiosis in *Buchnera* and their effects on host fitness have been the subject of several reviews in recent years, only a summary is presented here (Table 1).

The best-characterized S-symbionts have been found in *A. pisum* and in other members of subfamily Aphidinae [39,40]. The first S-symbionts described in the pea aphid were the gammaproteobacteria *Serratia symbiotica*, *Regiella insecticola*, and *Hamiltonella defensa* [68]. Since then, six additional S-symbionts have been found in aphids, five from phylum Proteobacteria, *Arsenophonus, Rickettsiella*, and *Fukatsia symbiotica* (previously known as X-type; Gammaproteobacteria) as well as *Ricketssia* and *Wolbachia* (Alphaproteobacteria), and one from phylum Firmicutes, *Spiroplasma* (Mollicutes) [31,39,40,62]. Of them, only *Wolbachia* and *Arsenophonus* have not been found in *A. pisum* so far (revised in [31]). Some S-symbiont genomes have been sequenced (Table 1), showing that they are already affected by the genome-reduction syndrome, since their genomes are smaller than those of their free-living relatives but larger than that of *Buchnera*. For example, the genome of *S. symbiotica* SAp (from *A. pisum*) is 2.57 Mb in length, quite shorter than that of the free-living *Serratia marcescens* (5.11 Mb). The result of their metabolic analysis is in accordance with their facultative status and helps in understanding some of the positive effects described for each association. An unexpected finding was that some facultative symbionts depend on *Buchnera* or another bacterium for some metabolic compounds, whose biosynthetic pathways are being pseudogenized, as is the case of *S. symbiotica* SAp [53].

The genome sequencing of *Buchnera* BCc in 2006 provided a striking result [28]. With only 362 protein coding genes, its genome represented a minimized set of genes able to support cellular life. It has conserved all the necessary genes for its own replication, transcription, and translation, as well as a simplified metabolic network to produce energy, and therefore could be considered as an autonomous cell in a particular environment [69]. However, it has partially lost its role in its symbiotic system because it is unable to synthesize tryptophan, as only the two first genes of the pathway, coding for anthranilate synthase, were present in a plasmid [70]. Prior to the publication of the *Buchnera* BCc genome, microscopic analyses had already shown that *Buchnera* BCc was not alone in the cedar aphid *C. cedri* [65], and it was proposed that it could be in the process of being replaced by the “healthier” coexisting *S. symbiotica* [28]. The sequencing of the *S. symbiotica* SCc genome proved that it contains the essential tryptophan genes lost in *Buchnera* BCc but has lost the anthranilate synthase genes preserved in *Buchnera* [56]. This complementation implies that anthranilate, the first metabolite in the pathway and synthesized by *Buchnera*, should go to *S. symbiotica* to be used as a precursor to synthesize tryptophan, which is then supplied to the three members of the consortium (i.e., *Buchnera*, *Serratia*, and *C. cedri*). This was the first described endosymbiotic consortium involving two co-obligate bacteria, *Buchnera* BCc and *S. symbiotica* SCc [56,70]. Almost at the same time, the genome of the facultative *S. symbiotica* SAp was published [53], confirming important differences in the two *S. symbiotica* lineages due to their obligate or facultative statuses, respectively.

The sequencing of a third *S. symbiotica* genome from the thuja aphid *C. tujafilina*, a close relative of *C. cedri*, provided a new surprise because *S. symbiotica* SCt was morphologically and genomically closer to SAp than to SCc [29,57]. The genome analysis indicated that it represents another intermediate stage in the accommodation process into the aphid-*Buchnera* system, and it still presents the whole tryptophan biosynthetic pathway. The sequencing of *S. symbiotica* STg from *Tuberolagnus salignus*, another member of subfamily Lachninae, revealed an even more degenerate genome (0.65 Mb) compared with that of *S. symbiotica* SCc (1.76 Mb) [55,56]. A revisiting to all *Buchnera* genomes from Lachninae aphids indicated that the obligate consortium between *Buchnera* and *S. symbiotica* was triggered by the ancient loss of the pathway for the biosynthesis of riboflavin in an ancestral *Buchnera* of this subfamily [45]. Since then, *S. symbiotica* has undergone specific genome reduction patterns in each lineage, and phylogenetic analyses indicate that the loss of the tryptophan gene has occurred convergently in the bacterial lineages present in *C. cedri* and *T. salignus*.

A critical issue in evolutionary biology is finding traits or organisms that provide evidence of the transition from one lifestyle to another. The discovery of *S. symbiotica* in different stages of the accommodation to intracellular life, and their comparison with free-living relatives, offered the opportunity of comparing bacteria under three different lifestyles—free-living, facultative, and obligate endosymbionts—and allowed for the dissecting of the genomic changes undergone in the process. The free-living species chosen for the comparison was *Serratia marcescens* Db11, from *Drosophila melanogaster* [71]. In addition to the four above-mentioned *S. symbiotica* strains (three co-obligate in the Lachninae and one facultative in *A. pisum*), the facultative strain SAf from *Aphis fabae* (subfamily Aphidinae) was also sequenced. With the largest *S. symbiotica* genome (3.58 Mb), *S. symbiotica* SAf can still grow in axenic conditions [72,73]. Although each lineage may undergo convergent decay and other individually relevant processes, what is clearly observed is a gradual decrease in genome size in the different stages of the integrative symbiotic process, from free-living to co-obligate, which was accompanied by gradual changes in other genome characteristics, including the GC content (from 52.1 to 20.9%), the number of coding genes (from 3398 to 495), as well as a gradual reduction in the number of copies of rRNA, tRNAs, and other non-coding RNA genes, revealing different levels of genome erosion. Other changes were not as clearly correlated with genome reduction but provided new clues on the intermediate stages of the process, which can be specific to the evolutionary momentum of each lineage. Thus, there is great enrichment of pseudogenes in the two facultative and two co-obligate strains, whereas the small *S. symbiotica* STs genome is almost deprived of them. Additionally, like other large endosymbiotic genomes, *S. symbiotica* SAf, SAp, and SCt display a great enrichment of mobile elements in comparison with the free-living counterpart, whereas in the smaller genomes of *S. symbiotica* SCc and SCt, there are no even traces of such elements, in congruence with similar-sized endosymbiotic genomes [74]. The increase in mobile elements in recently acquired facultative endosymbionts, with a great potential as recombination sites, and their progressive loss leading to their complete disappearance in obligate endosymbionts are implied in the abundance of chromosomal rearrangements detected between free-living and facultative symbiont genomes, opposite to the chromosomal stasis in long-term P-endosymbionts [75].

### 2.3. Replacement of Symbionts

The genome-reduction syndrome undergone by obligate endosymbionts may end compromising their corresponding symbiotic functions by the loss or pseudogenization of genes that are necessary for the association, unless the host or another symbiont can compensate for such functional losses [8]. As stated above, one solution is the establishment of an obligate symbiotic consortium, when the coexistence of both bacteria maintains a “healthy” system by establishing metabolic complementation. Another possibility is the replacement of the inefficient P-endosymbiont by a new intracellular bacterium that has not yet started losing essential functions, and can even be a source of novel metabolic capabilities to the host. However, despite the amount of partners that can coexist with *Buchnera*—either facultative or co-obligate—and its reduced genome, only two cases of *Buchnera* replacement have been documented up to now. Members of the Asian tribe Ceratiphidini (subfamily Ceratiphinae) lack *Buchnera*, which has been replaced by a yeast-like symbiont (YLS) [76]. The functional and evolutionary analysis of this eukaryotic symbiont revealed that it is vertically transmitted, has a broader metabolic repertoire than *Buchnera*, and thus can fulfill nutritional host needs [77]. The second case has been found very recently in members of genus *Geopemphigus* (Erisosmatinae: Fordini) [78], where *Buchnera* has been replaced by *Skilesia alterna*, a maternally transmitted bacterial symbiont from the phylum Bacteroidetes. As in the previous case, this new endosymbiont has retained biosynthetic pathways for essential amino acids and vitamins. Moreover, its 1.32 Mb genome and its 37.0% GC content indicates that the degenerative genome syndrome is underway.

The subfamily Lachninae is particular among aphids, not only because all its members seem to have established co-obligate associations with *Buchnera* but also because multiple replacements of the co-obligate symbiont have been documented [33,41,44,45,79,80,81]. As stated previously, in this subfamily, *Buchnera* holds highly reduced genomes and, probably because of the loss of the riboflavin pathway in the ancestor of the lineage, members of this subfamily depend on a second co-obligate endosymbiont to complement *Buchnera* functions [41,45,53,55,57]. So far, the analysis for the presence of endosymbiont bacteria in species representative of five tribes of this subfamily (Lachnini, Stomaphidini, Tramini, Tuberolachnini, and Eulachnini) indicates that, while most species host *S. symbiotica*, some are associated with other members of Enterobacteriaceae (summarized in [45])*. Fukatsia symbiotica* was found in some Lachnini and Eulachnini species; *Serratia marcescens*-like secondary symbiont (SMLSS), *Arsenophonus*, *Gilliamella*-like secondary endosymbiont (GLSS), and *Erwinia*-like bacteria in species belonging to Stomaphidini; *Sodalis*-like secondary symbiont (SLSS) and *Hamiltonella* in some species of Tuberolachnini and Eulachnini; and *Wolbachia* in Stomaphidini and Eulachini. The most parsimonious scenario to explain all these data is that a second endosymbiont, probably *S. symbiotica*, was established early in the ancestor of subfamily Lachninae, followed by at least six independent events of symbiotic replacements and, eventually, the recruitment of a third endosymbiont.

*Cinara* (Eulachnini) is the most diverse genus of the subfamily, and it has been broadly studied for the characterization and phylogenetic analysis of the resident symbiont accompanying *Buchnera* [41]. A recent study by Meseguer and coworkers supports the previous hypothesis of *S. symbiotica* present in the common ancestor of the genus, followed by its diversification and replacements in different clades, in eight cases by another *Serratia*. All the Enterobacteriaceae species detected previously, plus *Rickettsia*, *Regiella*, *Edwardsiella*, *Acinetobacter*, and an unnamed member of this family, were detected in at least one species. The authors did not find any association between the acquisition of a new symbiotic partner and the ecological expansion of the corresponding aphid hosts. For this reason, they proposed that the symbiotic succession would be driven by factors such as genome deterioration or competition between bacteria with similar metabolic capabilities. Morphology of the co-resident bacteria has been studied by FISH in some species, revealing a wide variety of cell shapes and tissue tropism for the same bacterial genus in different insect hosts. Shifts in tissue tropism could cause bacteriocyte arrangements within the bacteriome and stable internalization of the second symbiont in distinct bacteriocytes [45]. Once this happens, there is probably no turning back and the genome reduction accelerates until a genome similar to that found in *S. symbiotica* from *T. salignus* is generated [55].

## 3. The Case of Cicadas and Relatives: *Sulcia* and Its Multiple Partners

### 3.1. Consortia and Replacements in the Auchenorrhyncha

**Figure 1:**
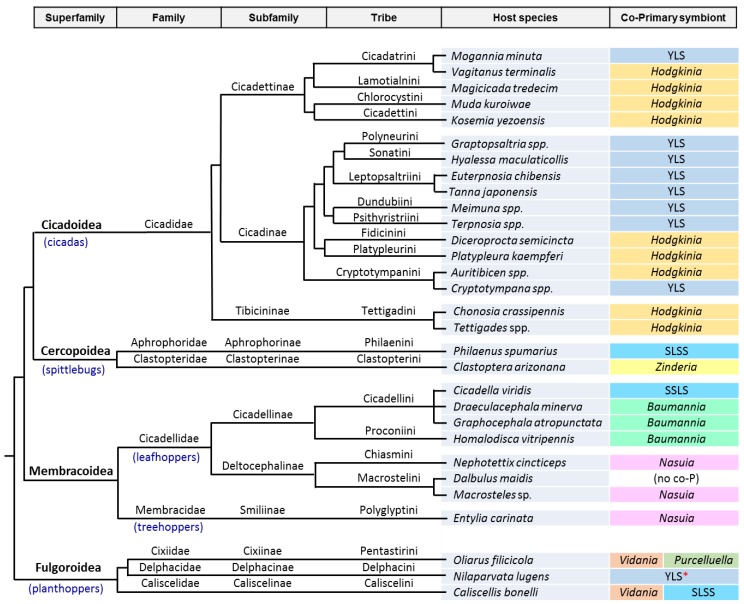
Schematic summary of the different clades of Auchenorrhyncha mentioned in the text and their corresponding co-primary endosymbionts that have been found in addition to *Sulcia* (except in the case labeled with an asterisk, where the YLS is replacing *Sulcia*). The evolutionary relationship of the clades is based on [104,105,106].

A second large group of sap-sucking insects of the order Hemiptera, extensively studied for the presence of P-endosymbionts, is the Auchenorrhyncha (Figure 1; Table 2). This suborder is composed of four superfamilies grouped in two main lineages: Cicadoidea (cicadas), Cercopoidea (spittlebugs), Membracoidea (leafhoppers and treehoppers), grouped in the cicadomorph clade, and Fulgoroidea (planthoppers). Xylem-feeding appears to be an ancestral character of all three modern cicadomorph lineages and has been retained in cicadas, spittlebugs, and a few primitive leafhoppers [10]. Buchner, and specially his student H. J. Müller, extensively studied this group of insects [82]. Their microscopic survey of hundreds of species allowed for the observation that most of them contain more than one symbiont, although they all shared a common one that was called at that time “a-symbiont.” Müller’s hypothesis was that the ancestor of the a-symbiont infected the ancestor of the Auchenorrhyncha before the split of the two main clades. He also proposed that the a-symbiont was joined, and sometimes replaced, by one or more additional symbiont types in different descendant host lineages, resulting in the current variety of associations. These hypotheses still hold based on metagenomic studies. This is now considered a perfect example of how the establishment of a symbiotic bacterial consortium can be at the origin of great evolutionary changes in the host’s lifestyle, while the genome degeneration of the consortium partners may end in the extinction and replacement of the more deteriorated and inefficient one, which is similar to what has also been described in aphids.

Müller’s a-symbiont was characterized by phylogenetic and FISH analyses in 2005 as a Bacteroidetes [83] and was given the name of *Sulcia muelleri*, the species name in his honor and the genus *Sulcia* after the pioneer symbiologist Karel Šulc. Its phylogenetic congruence with that of the corresponding hosts indicates that modern *Sulcia* are descendants of an ancient symbiont that was acquired by the ancestor of all Auchenorrhyncha members, at least 260 MYA. Soon after the description of this new endosymbiotic species, akin to what was found in *C. cedri* and almost at the same time, the gammaproteobacterium *Baumannia cicadellinicola* was discovered as a co-obligate endosymbiont with *Sulcia* in the leafhopper *Homalodisca vitripennis* [84]. However, that was just the beginning. Later on, the availability of genomes of many different *Sulcia* strains, as well as genomes of their variable partners in different xylem and phloem-feeding clades, revealed an endless story of alliances and replacements in the evolutionary history of *Sulcia* and its symbiotic fellows. We present only the tip of the iceberg of these complicated “family matters” (Figure 1).

At this time, up to 34 complete *Sulcia* genomes are available in GenBank, and in some cases their symbiotic partners have also been sequenced (Table 2). The analyzed *Sulcia* genomes have many features in common with what was found in *Buchnera*. An important difference is that, while *Buchnera* is the only P-endosymbiont in many aphid lineages, *Sulcia* has almost always been detected along with, and complemented by, one or more co-primary microorganisms. The *Sulcia* genomes are collinear [87,90], and the differences in their gene content imply a perfect metabolic complementation with the additional co-existing endosymbionts to provide their host with essential biomolecules lacking in their nutritionally deficient diet. Very often this involves the partial implementation of a given pathway in each of the partners. The ancestral *Sulcia* had an already streamlined genome, as deduced from the very small sizes of the extant *Sulcia* genomes that have been sequenced (from 157 to 285 kb).

Three betaproteobacterial species have been identified co-occurring with *Sulcia*: *Nasuia deltocephalinicola* in phloem-feeding leafhoppers of subfamily Deltocephalinae (family Cicadellidae) from whom it received the species name [98] but also of family Membracidae [96], *Zinderia insecticola* in many spittlebugs (Cercopoidea) [90], and *Vidania fulgoroideae* in planthoppers (Fulgoroidea) [99]. *Zinderia* and *Nasuia* (collectively named *BetaSymb* clade) are very closely related [94], which indicates that a common ancestor infected the lineage leading to Cicadomorpha early after the establishment of the endosymbiosis with *Sulcia*. *Vidania* appears to be a descendant of the ancient symbiont that infected the common ancestor of superfamily Fulgoroidea at least 130 MYA [82]. The genome reduction syndrome has been dramatic and these symbiotic relationships lead to some of the most highly reduced genomes sequenced to date (down to 112 kb for *Nasuia* ALF and PUNC, found in two leafhoppers of genus *Macrosteles*) [94,95]. These tiny genomes are below the minimal genome status because they have lost genes needed for the maintenance of a living cell (including DNA replication, transcription, and translation) [69,100]; therefore, even these essential functions must be performed in cooperation and shared by the joined symbiotic partners.

While *Sulcia*—like *Buchnera*—is rarely lost, the Beta-endosymbionts have been secondarily lost many times and, akin to what has been described in Lachninae aphids, in most cases they have been replaced by different “healthier” partners [8]; in other cases, a third partner joined the consortium to cope with the extreme genome degeneration of the two oldest co-primary endosymbionts (Figure 1). Thus, in the *BetaSymb* clade, *Zinderia* has been replaced by the alphaproteobacterium *Hodgkinia cicadicola* in cicadas (Cicadoidea) [94], and *Nasuia* has been replaced by *Baumannia* in subfamily Cicadellinae [84,91,92,101] and, although it has been retained in most analyzed members of the sister subfamily Deltocephalinae, it seems to have been lost in the corn leafhopper, *Dalbulus maidis* [93,102]. In the Fulgoroidea, *Vidania* and *Sulcia* have been found together with the gammaproteobacterium *Purcelliella pentastirinorum* in several planthoppers of family Cixiidae [82,97,99,103].

In many cases, the new allied endosymbiont has been recruited from the same clades that have been repeatedly found as pathogens or facultative symbionts in other sap-feeding insects, suggesting that they can be an environmental source for symbiont exchange and evolutionary reinvigoration when the P-endosymbiont cannot cope with its symbiotic function. As mentioned before, in aphids there are cases in which intracellular symbiotic yeasts have replaced *Buchnera*. Similarly, there are many cases in which YLS phylogenetically related to the entomopathogenic genus *Ophiocordyceps* (Ascomycota: Sordariomycetes: Hypocreales) have joined or replaced *Sulcia* and/or its bacterial partner. Thus, *Sulcia* has been replaced in some young lineages of Delphacidae planthoppers (Fulgoroidea) [107]; neither *Vidania* nor *Sulcia* were found in some young lineages of family Delphacidae where a vertically transmitted YLS was found [107,108]; and the same kind of YLS was found in some leafhoppers of subfamilies Deltocephalinae [109,110,111] and Ledrinae [112], whether replacing *Nasuia* or in an intermediate stage in which both *Nasuia* and the YLS coexist with *Sulcia*. In addition, many independent cases of *Hodgkinia* replacement have been found in several tribes of two cicadidae subfamilies; in fact, it is possible that repeated *Hodgkinia*-fungus and fungus–fungus replacements had occurred [85]. Located in the fat body of the insects, these YLS play an essential role in uric acid recycling [107,113]. It is worth mentioning that, in the cases of *Buchnera* replacement in aphids, the YLS serves a different endosymbiotic purpose [77]. As for common environmental and facultative symbiotic bacteria, several *Sodalis*-like symbionts have participated in replacements or tripartite associations in the Auchenorrhyncha. For example, in superfamily Cercopoidea, a replacement of *Zinderia* by a *Sodalis*-like symbiont or intermediate tripartite consortia have been described in spittlebugs of the tribe Philaenini [89,114]. In superfamily Fulgoroidea, a *Sodalis*-like symbiont has joined the association between *Sulcia* and *Vidania* in *Caliscelis bonelli* (Caliscelidae) [115]. In all the above-mentioned associations, each endosymbiont lives in its own bacteriocytes, and, in some cases, additional putative facultative symbionts have been also found [107,116,117,118].

Among all the diversity of endosymbiotic systems found in Auchenorrhyncha, the most widely studied correspond to cicadas, since it is in this superfamily that the most bizarre endosymbiont genomes have been found. For this reason, they deserve their own subsection in this review.

### 3.2. The Peculiarities of the Hodgkinia Genomes and Its Coexisting Interdependent Lineages within a Single Host

The genomes of different *Hodgkinia* strains have been sequenced, and every new genome analyzed provided new surprises. The first genome, from the P-endosymbiont of the glassy-winged sharpshooter *Diceroprocta semicincta* [119], had a GC base composition of 58.4%, very different from all other endosymbiont genomes sequenced at that time. Most strikingly, the genome annotation revealed that the UGA stop codon was reassigned to tryptophan in this bacterium. The same codon reassignment was later detected in the tiny genomes of *Zinderia* and *Nasuia* [90,94], even though they were AT-rich, ruling out the hypothesis that base composition is a root cause of codon reassignment. It is worth mentioning that the same genetic-code modification had been previously described in the reduced and AT-rich genomes of mycoplasmas [120] and some mitochondrial lineages [121], representing a remarkable example of evolutionary convergence. It has been proposed that this reassignment was triggered by the loss of release factor RF2 (encoded by *prf*B), whose function is to recognize this stop codon [119].

Nevertheless, what makes *Hodgkinia* strains extraordinary is their capability to present alternative interdependent lineages, with different genotypes and genome rearrangements, inside a given host. The coexistence of two cytologically distinct but metabolically interdependent *Hodgkinia* clades, with reciprocal patterns of gene loss and retention, was first detected in some cicadas of genus *Tettigades* [88]. Soon later, an impressive level of genome complexity was described in the longest-lived cicadas of genus *Magicicada* [86,122]. In each *Magicicada* species, the *Hodgkinia* genome is composed by many subgenomic circles of different size, with an extremely reduced gene density. Additionally, the same gene can be present in different circles, and not all circles are present in all *Hogkinia* cells within a single host. Similarly to other highly reduced endosymbiont genomes, and contrary to the first *Hodgkinia* genomes sequenced, most of these complex *Hodgkinia* genomes have a low GC content (e.g., among the 39 sequenced subgenomic circles of the *Hodgkinia* found in *M. neotredecim*, the GC content ranges from 21.9 to 42.4, with only three circles having a GC content above 35% [122]), an indication that a high GC content is not a general trait of this species. Remarkably, genome instability and expansion because of the increase of “junk DNA,” leading to the existence of subgenomic molecules with low coding capacity, are common in mitochondrial lineages from some plants, another example of evolutionary convergence. More recently, an astonishing level of complexity was detected when 19 different *Hodgkinia* genomes, isolated from only five specimens from diverse Chilean populations of *Tettigades* spp., were sequenced [87]. The results suggest that a single ancestral *Hodgkinia* lineage has split at least six independent times in this insect genus over the last 4 million years. Two to six *Hodgkinia* lineages can be found in each single host, and each lineage presents different genomes formed by subgenomic molecules. Each lineage genome contains a different set of genes and, most of the time, does not contain all genes needed for the symbiotic relationship nor the provision of essential informational bacterial functions (i.e., DNA replication, transcription, and translation). Therefore, these different lineages coexisting in a single host rely on each other to survive. Furthermore, different combinations of *Hodgkinia* lineages can be found in each host. The degenerative process leading to this amazing splitting phenomenon is progressive and has no way back, since it will be impossible to recover the genomic information that has been lost. The extreme degeneration of these genomes appears to indicate that this endosymbiont has reached a critical stage in genome erosion and could be close to collapse, which could be the cause of the large number and variety of replacements found in this superfamily of insects.

## 4. The Case of Mealybugs: Not a “Simple” Matryoshka Doll

### 4.1. A Surprising Nested Endosymbiotic System

Another kind of exceptional consortium was found in most mealybugs (Hemiptera: Sternorrhyncha: Pseudoccidae) of subfamily Pseudococcinae. Early symbiologists, including Buchner, explained that this symbiotic system was formed by bacteria embedded within mucous spherules [123]. By microscopic and molecular studies on the citrus mealybug *Planococcus citri*, von Dohlen and coworkers discovered that such vesicles were, in fact, a nested endosymbiotic system [124]. In these insects, the betaproteobacterium *Tremblaya princeps* harbors a gammaproteobacterium inside. No nested endosymbiotic consortia have been described outside this mealybugs’ subfamily up to now.

The inner gammaproteobacteria were initially considered as S-symbionts because they are polyphyletic [123]. However, genomic studies performed on several Pseudococcinae mealybugs revealed that they are co-primary, based on the intricate interdependence between both symbionts, involving not only metabolic but also informational functions. Several nested endosymbiotic consortia have been sequenced [125,126,127,128,129], revealing many commonalities among them that must also be considered quite unusual for obligate endosymbionts, although some of them are also shared with some *Hodgkinia* strains. Thus, the comparison among several *T. princeps* strains revealed a highly conserved genome architecture, as in other long-established P-endosymbionts [128], but they all have a GC-content higher than expected for a P-endosymbiont. Although all sequenced strains possess tiny genomes (137–144 kb), they have a reduced coding density and present repeated sequences evolving under concerted evolution, including a partial genomic duplication of the ribosomal operon and neighbor genes [130]. In fact, even though ribosomal genes and genes involved in the biosynthesis of essential amino acids constitute most of its genome, *T. princeps* depends on its inner partner to make up both ribosomes and essential amino acids [125,126].

**Figure 2:**
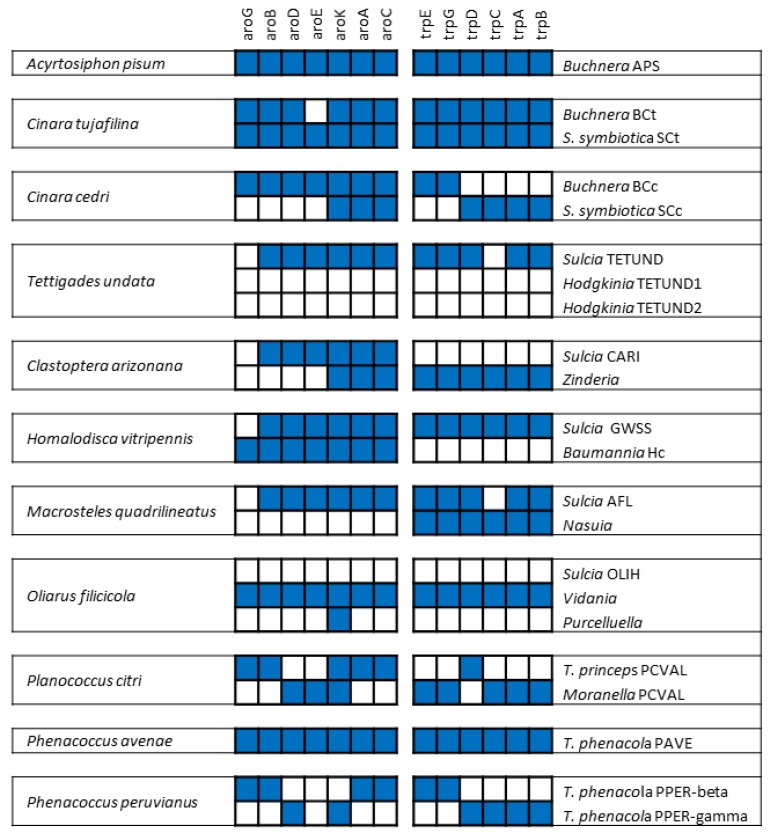
Diverse solutions implemented by different symbiotic systems for the biosynthesis of tryptophan from D-erythrose-4-phosphate, showing the complementation achieved in different consortia. Notice that *T. phenacola* PPER corresponds to a single chimeric genome.

While patchwork complementation for amino acid biosynthesis is a common feature of many endosymbiotic consortia (Figure 2), this was the first described case in which all energy sources must be provided by one of the symbiotic allies, similar to mitochondria in the eukaryotic cell. The preservation of two genes coding for channels associated with osmotic stress response (*msc*L and *yba*L) in the *T. princeps* genome was also surprising, because they have not been identified in other long-term P-endosymbionts. It was proposed that they might reflect a special requirement of nested endosymbiosis, and be involved in the exchange of molecules between both partners [127]. As for the inner gamma-endosymbiont, the maintenance of an almost-complete DNA recombination machinery, unusual among long-established endosymbionts, has been proposed to be involved in the concerted evolution of the duplicated loci in *T. princeps* [131].

Similarly to what was found in the other model consortia described in this review, different mealybug lineages have gone through a complex evolutionary history of independent acquisitions and replacements of different nested gamma-endosymbionts [128,129,132]. This is an indication that, even in this highly integrated symbiotic consortia, extensive symbiont turnover is possible, and maybe essential, for evolutionary reactivation of extremely degenerate genomes. Most of these newly acquired inner bacteria belong, once more, to the *Sodalis-like* clade, although they do not form a monophyletic group.

### 4.2. The Fist Chimeric Endosymbiont

Mealybugs are classified in subfamilies Pseudococcinae (the one described above) and Phenacoccinae. Based on phylogenetic studies, the betaproteobacterial ancestor of genus *Tremblaya* infected the mealybug ancestor before the split of both subfamilies [133]. As seen above, *T. princeps* evolving in most Pseudococcinae lineages were later on infected multiple times by different gammaproteobacteria. This was not the case of the sister species *Tremblaya phenacola* in the Phenacoccinae, which has always been described alone or has been replaced by different Bacteroidetes in some clades [22,134]. In coherence with this view of the need of a single P-endosymbiont in this subfamily, the highly reduced genome of *T. phenacola* PAVE, from *Phenacoccus avenae*, presented all genes needed to fulfill all essential endosymbiotic functions attributed to the beta-gamma consortia found in Pseudococcinae mealybugs [22]. Surprisingly, when the genome of the endosymbiont from the close relative *Phenacoccus peruvianus* was sequenced, it was found that nearly half of the genome contained sequences taxonomically affiliated to Gammaproteobacteria [135], even though the bacterium was named *T. phenacola* PPER based on its 16S rRNA gene [132]. Additionally, the genome could not be fully assembled, even using Nanopore sequencing (which allows for the sequencing of long DNA molecules, up to several hundred kb). In fact, the results obtained indicated the coexistence of different genome organizations, none of which corresponded to a full genome, favored by the presence of many repeated sequences. Although the possibility of lineage splitting has not been analyzed in this insect, its similarity to the case of *Hodgkinia* points to that. It seems that, at least in the lineage leading to *T. phenacola* PPER, a gammaproteobacterium entered the symbiotic association. The establishment of a nested consortium, similar to that found in the Pseudococcinae, cannot be discarded prior to the genomic fusion of the two co-occurring endosymbionts. The presence of a recombination machinery in the newly acquired gamma-endosymbiont must have triggered the loss of redundant genes, as well as the observed gene shuffling, leading to a genome in which a single copy of each gene has been preserved [135]. Once more, the phenomenon resembles what has been found in mitochondria, where cases of genome fusion and rearrangements caused by homologous recombination events have been described [136,137].

## 5. Concluding Remarks and Future Perspectives in the Field

In this review we have presented paradigmatic examples of endosymbiotic systems, from the very beginning of the intimate association to the most extreme cases of genome reduction, including the ultimate fate of the endosymbionts once they reach a limit stage of degeneration in which they cannot fulfill the host needs. We have described the transformations undergone by a free-living bacterium on becoming an intracellular endosymbiont. We know now that, despite the huge variability of systems found in nature to date, convergent solutions are achieved in most cases (e.g., for the joined biosynthesis of amino acids and vitamins in patchwork pathways; Figure 2). Furthermore, bacterial and fungal species from some widely distributed clades living in sympatry with the insect hosts can enter the symbiotic association, and can be used as copartners or even replace the first obligate endosymbiont. This explains that, for example, some *Sodalis*-like bacteria are found in a huge number of associations with species from all three model insect lineages cited in this review.

The complete knowledge of a symbiotic system requires a system biology approach in which, in addition to in silico predictions based on genomic analyses and phylogenetic information, transcriptomic, proteomic, and metabolomic analyses are also needed. Some studies using these new approaches have been already performed [21,22,138,139,140,141]. For sure, further studies will come in the near future, allowing for the dissection of insect–endosymbiont interactions to disentangle the convergent solutions achieved in the different systems for host/endosymbiont(s) metabolic complementarity and collaboration.

From an applied point of view, the integration of all knowledge about these symbiotic systems will allow for the determination of the best conditions for laboratory culture of intracellular bacteria with reduced genomes. It will then be possible to design experimental approaches in which the bacteria can be manipulated and, through the elimination of non-essential genes, resulting in a simplified natural cell. There are other approaches of broad interest in synthetic biology, such as the possibility to generate an adequate chassis in which genetic modules are added for the performance of a function of interest, and the always ambitious goal of, using all this information, designing new semisynthetic cells with minimized genomes that support life with alternative minimal metabolisms.

## Figures and Tables

**Table 1:** List of *Buchnera* symbiotic partners that have been identified in different aphid lineages. The genomes of a few of them have been sequenced, and their characteristics are also presented. The classification of Aphididae follows [36].

Co-Symbiont: Bacterium (Class)	Aphid Subfamily: Tribe	Host Examples (genus)	Sequenced Genome (Host Strain)	Genome Size (Mb)	GC (%)	CDS	Refs.
*Arsenophonus* (γ-proteobacteria)	Aphidinae: Aphidini	*Aphis, Hyalopterus, Melanaphis*					[37,38,39,40]
*Acinetobacter*	Lachninae: Eulachnini *	*Cinara*					[41]
(γ-proteobacteria)	Lachninae: Stomaphidini *	*Stomaphis*					[42,43]
*Erdwardsiella*	Lachninae: Eulachnini *	*Cinara*					[41]
(γ-proteobacteria)							
*Erwinia*-like symbiont	Aphidinae: Aphidini	*Hyalopterus*					[39]
(γ-proteobacteria)	Lachninae: Eulachnini *	*Cinara*					[41]
GLSS (γ-proteobacteria)	Lachninae: Stomaphidini *	*Stomaphis*					[44,45]
*Hamiltonella defensa* (γ-proteobacteria)	Aphidinae: Macrosiphi	*Acyrthosiphon, Myzus Macrosiphon, Sitobion*	*A. pisum* 5AT	2.17	40.5	2,158	[40,46,47]
	Aphidinae: Aphidini	*Aphis, Hyalopterus*					[39,40,48]
	Lachninae: Tuberolachnini *	*Nippolachnus*					[42]
	Lachninae: Eulachnini *	*Eulachnus, Cinara*					[41,42,44]
*Regiella insecticola* (γ-proteobacteria)	Aphidinae: Macrosiphini	*Acyrthosiphon, Myzus Macrosiphum, Sitobion*	*A. pisum* LSR1	2.07	42.5	1,769	[40,46,49]
	Aphidinae: Aphidini	*Aphis*					[40,46,48]
	Lachninae: Eulachnini *	*Cinara*					[41]
*Ricketsiella viridis* (γ-proteobacteria)	Aphidinae: Macrosiphini	*Acyrthosiphon*	*A. pisum* RA04	1.6	39	1,378	[35,50]
*Rickettsia* (α-proteobacteria)	Aphidinae: Macrosiphini	*Acyrthosiphon, Uroleucon*					[40,48,51]
	Aphidinae: Aphidini	*Aphis*					[40,48,51]
	Lachninae: Eulachnini *	*Cinara*					[41]
*Serratia symbiotica* (γ-proteobacteria)	Aphidinae: Macrosiphini	*Acyrthosiphon, Myzus, Macrosiphum, Sitobion, Uroleucon*	*A. pisum* TUC	2.57	52.1	2,098	[40,46,52,53]
	Aphidinae: Aphidini	*Aphis, Rhopalosiphum, Hyalopterus*	*A. fabae* CWBI-2.3	3.58	52.1	3,398	[39,40,46,48,54]
	Lachninae: Lachnini *	*Pterochloroides, Lachnus*					[45]
	Lachninae: Stomaphidini *	*Stomaphis*					[42,43]
	Lachninae: Tramini *	*Trama*					[45]
	Lachninae: Tuberolachnini *	*Tuberolachnus*	*T. salignus* STs	0.65	20.9	495	[55]
	Lachninae: Eulachnini *	*Cinara*	*C. cedri* SCc	1.76	29.2	677	[41,56]
			*C. tujafilina* SCt-VCL	2.49	52.2	1,601	[57]
SLSS (γ-proteobacteria)	Lachninae: Tuberolachnini *	*Nippolachnus*					[42]
	Lachninae: Eulachnini *	*Eulachnus, Cinara*					[33,41,42,45]
SMLSS (γ-proteobacteria)	Aphidinae: Macrosiphini	*Acyrthosiphon, Sitobion*					[58]
	Aphidinae: Aphidini	*Rhopalosiphum*					[59]
	Lachninae: Stomaphidini *	*Stomaphis*					[42]
*Spiroplasma* (Mollicutes)	Aphidinae: Macrosiphini	*Acyrthosiphon*					[60]
	Aphidinae: Aphidini	*Aphis*					[40,48]
*Wolbachia* (α-proteobacteria)	Aphidinae: Macrosiphini	*Sitobion, Macrosiphum, Aulacorthum, Pentalonia*					[61,62,63]
	Aphidinae: Aphidini	*Aphis, Aphis (Toxoptera)*					[64]
	Chaitophorinae: Siphini	*Sipha*					[62]
	Eriosomatinae: Fordini	*Baizongia*					[62]
	Neophyllaphidinae	*Neophyllaphis*					[62]
	Lachninae: Stomaphidini	*Stomaphis*					[43]
	Lachninae: Eulachnini	*Cinara*					[62,65]
*Fukatsia symbiotica* (X-type)	Aphidinae: Macrosiphini	*Acyrthosiphon*					[66,67]
(γ-proteobacteria)	Lachninae: Lachnini *	*Maculolachnus*					[42,44,45]
	Lachninae: Eulachnini *	*Cinara*					[41,44,45]

* Lifestyle co-obligate with *Buchnera*; the rest are facultative. SLSS: *Sodalis*-like Secondary symbiont. SMLSS: *Serratia marcescens*-like secondary symbiont. GLSS: *Gilliamella*-like secondary symbiont.

**Table 2:** Genomes of P-endosymbionts of Auchenorrhyncha that have been completely sequenced to date. The host species are ordered as in Figure 1.

Insect host	P-endosymbiont	Genome size (kb)	GC (%)	CDS	Ref.
*Mogannia minuta*	*Sulcia* SMMOGMIN	243,55	22.30	220	[85]
*Vagitanus terminalis*	*Sulcia* SMVAGTER	245,30	22.70	227	[86]
	*Hodgkinia* HCVAGTER	353	30.0	nd	
*Magicicada tredecim*	*Sulcia* SMMAGTRE	268,54	22.70	224	[85]
	*Hodgkinia* HCMAGTRE	1571	29.1	252	
*Muda kuroiwae*	*Sulcia* SMMUDKUR	266,95	22.60	248	[85]
	*Hodgkinia* HCMUDKUR	909	27.1	nd	
*Kosemia yezoensis*	*Sulcia* SMKOSYEZ	244,20	22.80	221	[85]
	*Hodgkinia* HCKOSYEZ	1863	30.0	nd	
*Graptopsaltria bimaculata*	*Sulcia* SMGRABIM	271,62	22.60	253	[85]
*Graptopsaltria nigrofuscata*	*Sulcia* SMGRANIG	271,57	22.60	253	[85]
*Hyalessa maculaticollis*	*Sulcia* SMHYAMAC	272,58	22.50	249	[85]
*Euterpnosia chibensis*	*Sulcia* SMEUTCHI	273,71	22.60	257	[85]
*Tanna japonensis*	*Sulcia* SMTANJAP	278,30	22.50	256	[85]
*Meimuna iwasakii*	*Sulcia* SMMEIIWA	272,32	22.60	253	[85]
*Meimuna kuroiwae*	*Sulcia* SMMEIKUR	271,07	22.60	253	[85]
*Meimuna opalifera*	*Sulcia* SMMEIOPA	271,56	22.60	252	[85]
*Meimuna oshimensis*	*Sulcia* SMMEIOSH	270,60	22.60	253	[85]
*Terpnosia nigricosta*	*Sulcia* SMTERNIG	273,63	22.70	256	[85]
*Terpnosia vacua*	*Sulcia* SMTERVAC	273,80	22.60	256	[85]
*Diceroprocta semicincta*	*Sulcia* SMDSEM	276,98	22.60	242	[85]
	*Hodgkinia* Dsem	144	58.4	169	
*Platypleura kaempferi*	*Sulcia* SMPLAKAE	268,04	22.50	248	[85]
	*Hodgkinia* HCPLAKAE	349	47.9	nd	
*Auritibicen bihamatus*	*Sulcia* SMAURBIH	276,77	22.80	256	[85]
	*Hodgkinia* HCAURBIH	474	45.0	nd	
*Auritibicen japonicus*	*Sulcia* SMAURJAP	278,18	22.80	259	[85]
	*Hodgkinia* HCAURJAP	438	45.8	nd	
*Cryptotympana atrata*	*Sulcia* SMCRYATR	273,23	22.70	252	[85]
*Cryptotympana facialis*	*Sulcia* SMCRYFAC	270,78	22.70	238	[85]
*Chonosia crassipennis*	*Hodgkinia* CHOCRA	149	38.7	170	[87]
*Tettigades limbata*	*Hodgkinia* TETLIM1	145	45.4	130	[87]
	TETLIM2	131	45.1	73	
	TETLIM3	128	47.8	50	
	TETLIM4	126	47.2	47	
	TETLIM5	122	45.8	39	
*Tettigades auropilosa*	*Hodgkinia* TETAUR	126	46.3	117	[87]
*Tettigades chilensis*	*Hodgkinia* TETCHI1a	130	44.9	163	[87]
	TETCHI1b	129	44.8	156	
	TETCHI2	117	45.8	115	
	TETCHI4	106	45.6	114	
*Tettigades ulnaria*	*Hodgkinia* TETULN	150	46.4	170	[87]
*Tettigades undata*	*Sulcia* TETUND	270,03	23.00	247	[88]
	*Hodgkinia* TETUND1	134	46.8	121	
	TETUND2	141	46.2	140	
*Tettigades undata*	*Hodgkinia* TETLON1	133	47.7	104	[87]
	TETLON2a	140	46.5	128	
	TETLON2b	137	46.7	109	
*Philaenus spumarius*	*Sulcia* PSPU	285,35	20.90	257	[89]
*Clastoptera arizonana*	*Sulcia* CARI	276,51	21.10	246	[90]
	*Zinderia*	209	13.5	206	
*Draeculacephala minerva*	*Sulcia* DMIN	243,93	22.50	226	[91]
	*Baumannia*	636 + 3.5	31.6	517 + 5	
*Graphocephala atropunctata*	*Sulcia* BGSS	244,62	22.50	227	[92]
	*Baumannia*	759	39	669	
*Homalodisca vitripennis*	*Sulcia* GWSS	245,53	22.40	227	[84]
	*Baumannia*	686	33.2	595	
*Nephotettix cincticeps*	*Sulcia* NC	192,24	23.70	176	U
*Dalbulus maidis*	*Sulcia* ML	190,41	24.10	187	[93]
*Macrosteles quadrilineatus*	*Sulcia* ALF	190,73	24.00	188	[94]
	*Nasuia*	112	17.1	138	
*Macrosteles quadripunctulatus*	*Sulcia* PUNC	190,66	24.00	181	[95]
	*Nasuia*	112	16,6	138	
*Entylia carinata*	*Sulcia* ENCA	218,03	23.00	198	[96]
	*Nasuia*	144.6	15.2	159	
*Oliarus filicicola*	*Sulcia* OLIH	156,58	24.90	152	[97]
	*Vidania*	136	18.2	154	
	*Purcelluella*	480	21.2	431	

U: Unpublished; nd: no determined.
